# Identification of Potential Factors for the Promotion of Fucoxanthin Synthesis by Methyl Jasmonic Acid Treatment of *Phaeodactylum tricornutum*

**DOI:** 10.3390/md22010007

**Published:** 2023-12-20

**Authors:** Hao Liu, Yawen Chen, Heyu Wang, Yaxuan Huang, Ying Hu, Yuxiang Zhao, Yifu Gong

**Affiliations:** 1 Key Laboratory of Marine Biotechnology of Zhejiang Province, School of Marine Sciences, Ningbo University, Ningbo 315200, China; hao_liu@biotranstech.com (H.L.); 216004690@nbu.edu.cn (Y.C.); 2 College of Food and Pharmaceutical Sciences, Ningbo University, Ningbo 315200, China; wangheyu@nbu.edu.cn; 3 Institute of Bioengineering, Biotrans Technology Co., Ltd., Shanghai 201500, China; 4 United New Drug Research and Development Center, Biotrans Technology Co., Ltd., Changsha 410000, China; yaxuan_huang@biotranstech.com (Y.H.); ying_hu@biotranstech.com (Y.H.)

**Keywords:** carotenoid synthesis pathway, fucoxanthin, methyl jasmonic acid, *Phaeodactylum tricornutum*, PHATRDRAFT_54800, PHATRDRAFT_20677

## Abstract

Fucoxanthin, a vital secondary metabolite produced by marine diatoms, has great economic value and research potential. However, its popularization and application have been greatly restricted due to its low content, difficult extraction, and high production cost. Methyl jasmonic acid (MeJA) exerts similar inductive hormones in the growth and development as well as metabolic processes of plants. In *Phaeodactylum tricornutum* (*P. tricornutum*), MeJA treatment can increase fucoxanthin content. In this study, the effects of different concentrations of MeJA on the cell growth and the fucoxanthin content of *P. tricornutum* were explored. Meanwhile, this study used high-throughput sequencing technology for transcriptome sequencing of *P. tricornutum* and subsequently performed differential gene expression analysis, gene ontology (GO) enrichment analysis, Kyoto Encyclopedia of Genes and Genomes (KEGG) enrichment analysis, and weighted gene co-expression network analysis (WGCNA) for screening the hub genes for the promotion of fucoxanthin synthesis with MeJA-treated *P. tricornutum*. On this basis, the functions of the hub genes for the promotion of fucoxanthin synthesis with MeJA-treated *P. tricornutum* were further analyzed. The results revealed that the carotenoid synthesis-related genes PHATRDRAFT_54800 and PHATRDRAFT_20677 were the hub genes for the promotion of fucoxanthin synthesis with MeJA-treated *P. tricornutum*. PHATRDRAFT_54800 may be a carotenoid isomerase, while PHATRDRAFT_20677 may be involved in the MeJA-stimulated synthesis of fucoxanthin by exerting the role of SDR family NAD(P)-dependent oxidoreductases.

## 1. Introduction

*Phaeodactylum tricornutum* (*P. tricornutum*) is an important unicellular marine microalga that possesses a variety of active substances needed for life-sustaining activities, including unsaturated fatty acids, vitamins, polysaccharides, sterols, carotenoids, etc., [1]. With the characteristics of rapid growth, easy accessibility, and simple cultivation, *P. tricornutum* also has strong growth adaptability, for it is highly adaptable to temperature, salinity, light intensity, and pH [2]. In recent years, domestic and international studies have confirmed the potential application of *P. tricornutum* in biomedicine, health care, and functional food development [3].

*P. tricornutum* contains light-assisted synthetic pigments such as fucoxanthin, and it is a potential resource for the production of high-quality fucoxanthin [4]. It has been shown that the fucoxanthin content of *P. tricornutum* is higher than that of macroalgae [5]. Therefore, *P. tricornutum* can be used as a model organism to analyze the fucoxanthin synthesis pathway. As a carotenoid enriched in *P. tricornutum* with a unique molecular formula, fucoxanthin is an essential secondary metabolite produced by marine diatoms [6]. In marine macro- and micro-algae, there is a large amount of fucoxanthin, accounting for approximately 10% of total carotenoids [7]. In plant thylakoids, fucoxanthin binds to chlorophyll a to form the fucoxanthin-chlorophyll protein complex and participates in photosynthesis in plants as well as algae by capturing light and transferring energy [8]. In the application of biotechnology, fucoxanthin can perform various biological functions such as anti-tumor, anti-inflammatory, weight loss, antioxidant, regulating blood glucose level, and improving immunity, thus bringing good economic benefits in practical applications [8]. Some researchers have found that the fucoxanthin content in plant thylakoids can be significantly increased under appropriate growth environments and inducer treatment conditions [9]. Methyl jasmonic acid (MeJA) is a critical signaling substance in plants that plays a role similar to that of an inductive hormone in plant growth, development, and metabolism [10]. When exogenous MeJA enters plant cells, it can be hydrolyzed to jasmonic acid by enzyme action, which binds to free amino acids and then participates in the physiological and metabolic processes of plants [11].

A report by McGee D. et al. has determined the influence of MeJA on the diatom’s cellular physiology in the marine diatom *Stauroneis* sp. by shotgun proteomics and found that the signal transduction cascades lead to increased lipid and pigment content [12]. Currently, no related study has been published to our knowledge to increase the fucoxanthin content in algal cells by MeJA-treated *P. tricornutum*. This study explored the effects of different concentrations of MeJA on the cell growth and the fucoxanthin content of *P. tricornutum*. At the same time, this study used high-throughput sequencing technology to sequence the transcriptome of *P. tricornutum* and subsequently performed differential gene expression analysis, gene ontology (GO) enrichment analysis, Kyoto Encyclopedia of Genes and Genomes (KEGG) enrichment analysis, and weighted gene co-expression network analysis (WGCNA). Finally, the function of key genes for the promotion of fucoxanthin synthesis with the MeJA-treated *P. tricornutum* was analyzed to probe into the specific molecular mechanism of how fucoxanthin synthesized related genes with the regulation of *P. tricornutum*.

## 2. Results

### 2.1. Effect of MeJA on Cell Growth and Fucoxanthin Content of P. tricornutum

**Figure 1:**
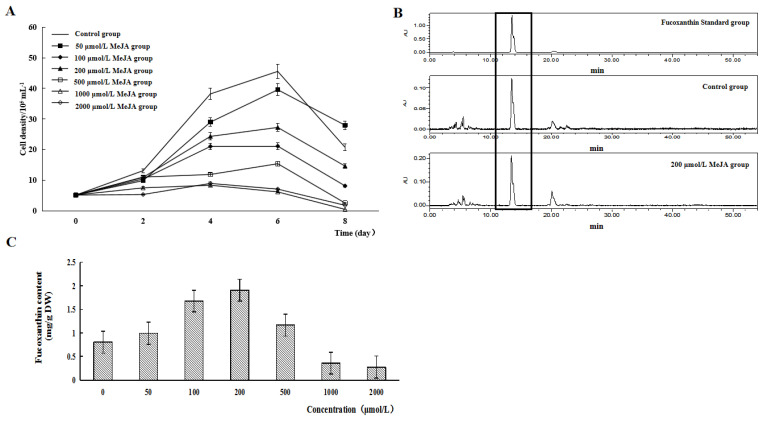
Effect of MeJA on the cell growth and the fucoxanthin content of *P. tricornutum*. (**A**) Cell growth curves of *P. tricornutum* in the control group and the MeJA group with different concentrations; (**B**) HPLC plots of the fucoxanthin standard group, the control group, and the 200 μmol/L MeJA group; (**C**) Fucoxanthin content in per gram of dry weight algae cells of *P. tricornutum* after treatment with different concentrations of MeJA.

With the prolongation of incubation time, the growth volume of the *P. tricornutum* cells was basically increased, and the algal cells reached the maximum biomass on the sixth day of incubation and then decreased. On the sixth day of culture, the algal biomass of different MeJA groups was less than that of the control group, and the algal biomass of the high-concentration MeJA (500–2000 μmol/L) was less than that of the low-concentration MeJA (0–200 μmol/L) (Figure 1A).

HPLC results signaled that the fucoxanthin standard group peaked at 13.5 min, while both the control and 200 μmol/L MeJA groups peaked at around 13.5 min (Figure 1B). Therefore, this study located the main peak that was highly similar to the fucoxanthin standard and subsequently detected fucoxanthin content in the control and MeJA-treated groups. In addition, the fucoxanthin content per gram of dry-weight algae cells showed a trend of increasing and then decreasing with the gradual increase in MeJA concentration. When the MeJA concentration was 50–500 μmol/L, the fucoxanthin content per gram of dry-weight algae cells was higher than that of the control group. When the MeJA concentration was 200 μmol/L, the highest fucoxanthin content per gram of dry-weight algae cells was found (Figure 1C). Therefore, the following studies used MeJA at a concentration of 200 μmol/L to treat *P. tricornutum* for transcriptome sequencing.

### 2.2. Differential Gene Expression Analysis

**Figure 2:**
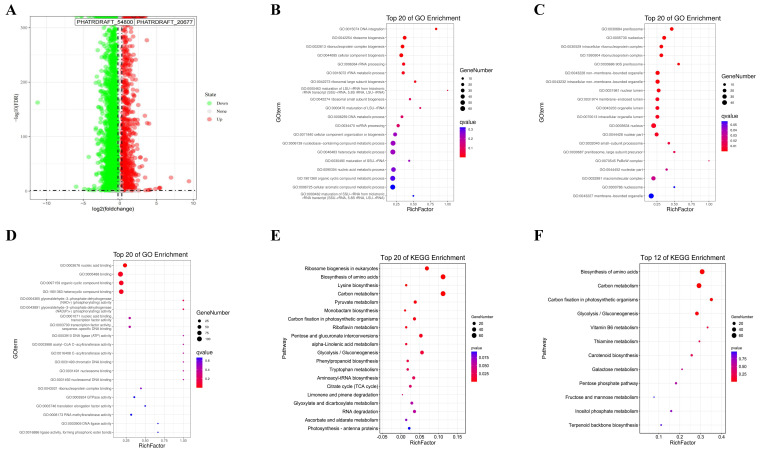
Differential gene expression analysis. (**A**) Volcano plots of expression profiles of the control and the MeJA groups. (**B**) Bubble chart of the GO-BP enrichment analysis of DEGs in the control and the MeJA groups. (**C**) Bubble chart of GO-CC enrichment analysis of DEGs in the control and the MeJA groups. (**D**) Bubble chart of GO-MF enrichment analysis of DEGs in the control and the MeJA groups. (**E**) Bubble chart of KEGG enrichment analysis of DEGs in the control and the MeJA groups. (**F**) Bubble chart of KEGG enrichment analysis of DEGs in the fucoxanthin synthesis-related pathway in the control and MeJA groups.

A total of 321 up-regulated and 925 down-regulated genes were screened through differential gene expression analysis (Figure 2A). In this study, the top 20 GO enrichments of differentially expressed genes (DEGs) included biological process (BP), cellular component (CC), and molecular function (MF). DEGs were associated with BPs such as DNA integration, ribosome biogenesis, ribonucleoprotein complex biogenesis, cellular component biogenesis, rRNA processing, rRNA metabolic process, and ribosomal large subunit biogenesis (Figure 2B). DEGs-enriched CCs were mainly preribosome, nucleolus, intracellular ribonucleoprotein complex, ribonucleoprotein complex, 90S preribosome, non-membrane-bounded organelle, intracellular non-membrane-bounded organelle, nuclear lumen, membrane-enclosed lumen, organelle lumen, intracellular organelle lumen, nucleus, and nuclear part (Figure 2C). MF included nucleic acid binding, organic cyclic compound binding, and heterocyclic compound binding (Figure 2D).

KEGG enrichment analysis pointed out that DEGs were mainly enriched in the pathways of ribosome biogenesis in eukaryotes, biosynthesis of amino acids, lysine biosynthesis, and carbon metabolism (Figure 2E). All metabolic pathways associated with fucoxanthin were further searched by using the KEGG mapper tool in the KEGG metabolic pathway database. A total of 12 metabolic pathways related to fucoxanthin synthesis were found, including biosynthesis of amino acids, carbon metabolism, carbon fixation in photosynthetic organisms, glycolysis/gluconeogenesis, vitamin B6 metabolism, thiamine metabolism, carotenoid biosynthesis, galactose metabolism, pentose phosphate pathway, fructose and mannose metabolism, inositol phosphate metabolism, and terpenoid backbone biosynthesis (Figure 2F).

### 2.3. WGCNA

**Figure 3:**
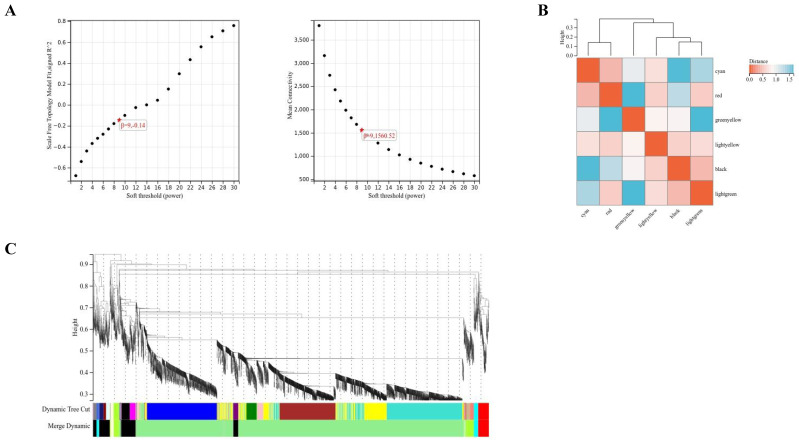
WGCNA. (**A**) Correlation plots between soft thresholds and scale-free networks through the construction of the WGCNA network. (**B**) Eigengene adjacency heatmap. (**C**) Cluster dendrogram of DEGs.

A weighted gene co-expression regulatory network was constructed with the soft threshold set to 9 (Figure 3A). Clustering was performed based on the dissimilarity metric of the topological overlap matrix (1-TOM), which divided the tree into six gene modules according to the dynamic tree cut. Eigengene adjacency heatmap demonstrated the clustering relationship between WGCNA modules and modules (Figure 3B,C). The screening condition for hub genes within a module was |kME| > 0.9.

**Figure 4:**
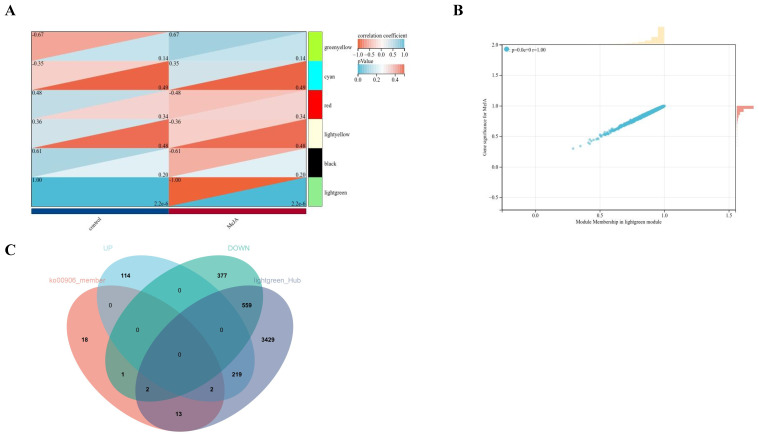
Relationship between gene modules and phenotypes. (**A**) Correlation heatmap between individual modules and phenotypes. (**B**) Correlation plot between phenotypes and light-green module hub genes. (**C**) Venn diagrams.

The phenotypic features included in this study were in the control and MeJA groups. Based on the feature vectors of each module, the correlation between these modules was calculated separately and plotted as intramodular connectivity. It was found that the module associated with MeJA treatment was light-green, in which various hub genes were present (Figure 4A). In addition, this study found that the MeJA treatment phenotype had the highest correlation with the light-green module (*R* = 1, *p* < 0.001) (Figure 4B). Finally, this study took the intersection of DEGs, carotenoid synthesis pathway (ko00906 carotenoid biosynthesis) genes, and members of the light-green module hub genes and discovered four overlapping genes, of which two were significantly upregulated (PHATRDRAFT_54800 and PHATRDRAFT_20677), and two genes were significantly down-regulated (*PDS1* and PHATRDRAFT_15806) (Figure 4C).

### 2.4. Analysis of Hub Genes for the Promotion of Fucoxanthin Synthesis in MeJA-Treated P. tricornutum

**Figure 5:**
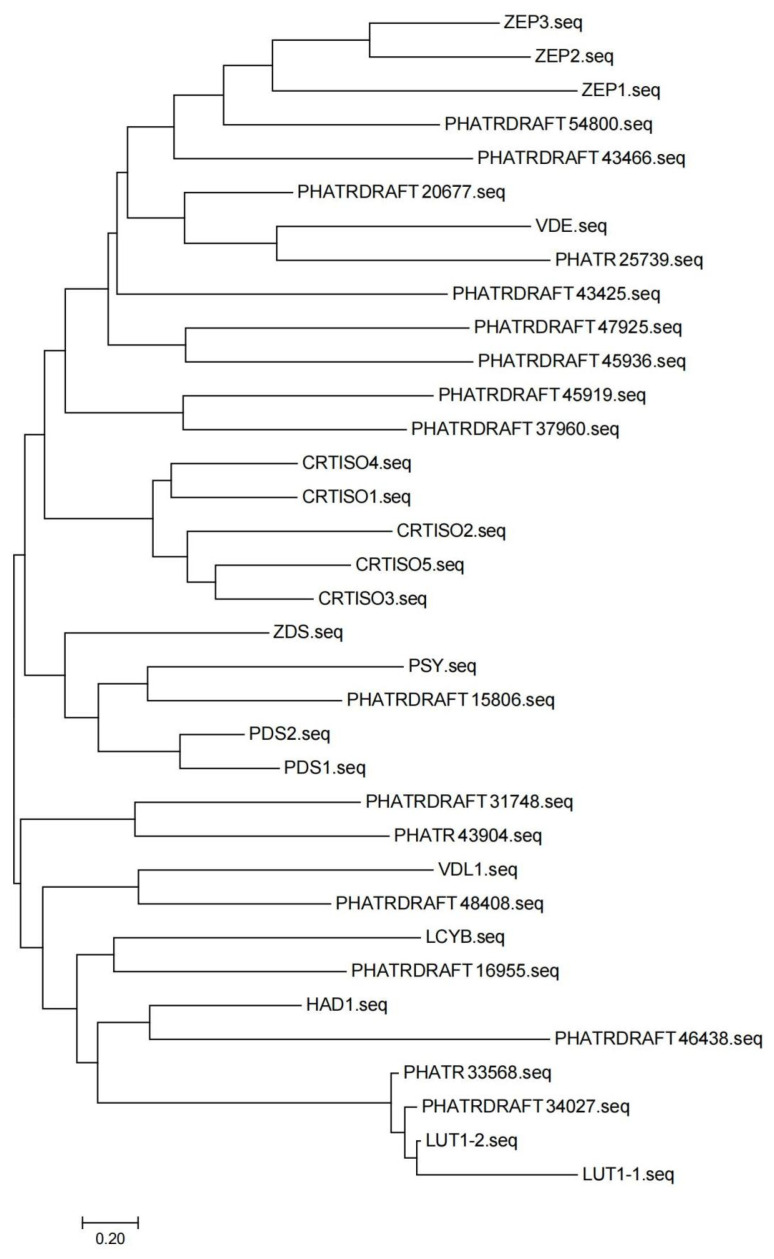
Molecular evolutionary tree of carotenoid synthesis-related genes in *P. tricornutum*.

From the above findings, four DEGs related to the synthesis pathway of fucoxanthin were obtained, two of which were up-regulated and two of which were down-regulated. According to previous studies of MeJA treatment of *P. tricornutum* [9], the functional hub genes should be the up-regulated genes (PHATRDRAFT_54800 and PHATRDRAFT_20677). As shown in the molecular evolutionary analysis tree, PHATRDRAFT_54800 clustered with the zeaxanthin epoxidase (Zep) family of genes, suggesting that PHATRDRAFT_54800 may be functionally similar to *Zep*. The closest gene to PHATRDRAFT_20677 was *VDE*, but its clustering level was higher than that of PHATRDRAFT_54800 and *Zep*. Therefore, it was difficult to determine the functional similarity between PHATRDRAFT_20677 and *VDE*, which deserved further analysis in the future (Figure 5). BLAST sequence alignment results revealed that the PHATRDRAFT_20677 was more similar to the SDR family NAD(P)-dependent oxidoreductases, and the top 10 similar sequences are shown in Table S1.

## 3. Discussion

MeJA is a compound derived from linolenic acid with a cyclopentanone group as its basic structure. MeJA, as a signaling substance in plants, regulates plant growth and development and participates in the production and accumulation of secondary metabolites during plant growth and development [13]. When plants are treated with exogenous MeJA, MeJA enters plant cells, hydrolyzes to jasmonic acid under the action of enzymes, and forms complexes, which in turn affect the translation of related proteins and induce the expression of genes in the synthesis pathway of plant secondary metabolites. Finally, a plant stress response is produced, which affects the synthesis of plant secondary metabolites and regulates the growth and metabolism of plants [14,15]. MeJA regulates the expression of genes related to plant development and self-defense systems and plays a signaling role in plant secondary metabolism [16]. Arano-Varela H. and others reported that MeJA had an effect on biomass and verbascoside production in cell suspension cultures of *B. cordata*. When 200 μM MeJA was used in the experiment, the biomass accumulation seemed to trade off with verbascoside production, which may be caused by the effect of MeJA on the amplification of its signal transduction pathway [17]. By utilizing MeJA to treat cabbage, Baek MW’s team has found that MeJA is effective in increasing the content of thioglucosides and total flavonoids, as well as improving the antioxidant properties of other vegetables such as cabbage [18]. Luo H’s team has reported that 0.5 μM MeJA treatment of germinated corn kernels could affect the carotenoid biosynthesis genes, which is favorable to promote carotenoid synthesis and increase proline accumulation [19].

As an inducer, MeJA can regulate the expression of genes related to plant development and the self-defense system and plays a role in signal transmission in the secondary metabolism of plants [20]. This experiment used different concentrations of MeJA to treat *P. tricornutum*, and it was found that with the prolongation of the incubation time, the growth volume of the *P. tricornutum* cells was basically increased, and the algal cells reached the maximum biomass at the sixth day of incubation and then decreased. In addition, the fucoxanthin content per gram of dry-weight algae cells tended to increase and then decrease with the gradual increase in MeJA concentration. When the MeJA concentration was 200 μmol/L, the fucoxanthin content per gram of dry-weight algae cells was the highest, which was 139% higher than that in the control group. This experiment indicated that the biosynthesis of fucoxanthin in *P. tricornutum* had a certain preference for the concentration of MeJA, and a high fucoxanthin content was obtained by treating *P. tricornutum* with 200 μmol/L MeJA. In addition, through the biocurves of the algal cells, it was suggested that the cell growth of *P. tricornutum* was inhibited whether *P. tricornutum* was treated with high or low concentrations of MeJA. This phenomenon may be caused by the duality of the inducers. In other words, high concentration inducers inhibited algal cells, and low concentration inducers did not work on algal cell growth. In contrast, the biomass of *P. tricornutum* treated with a high concentration of MeJA was significantly higher than that of *P. tricornutum* treated with a low concentration of MeJA, indicating that the inhibitory effect of MeJA on the algae was the dominant factor in the high concentration of MeJA.

In order to investigate the biosynthesis mechanism of MeJA on *P. tricornutum*, this study further explored the gene information in the synthesis pathway of fucoxanthin using transcriptomics methods. *P. tricornutum* was treated with 200 μmol/L MeJA and analyzed using transcriptome sequencing. A total of 321 up-regulated genes and 925 down-regulated genes were screened by differential gene expression analysis. From the results of GO enrichment analysis and KEGG enrichment analysis, DEGs were mainly enriched in ribosomal biogenesis, ribosome biogenesis in eukaryotes, biosynthesis of amino acids, lysine biosynthesis, carbon metabolism, and other pathways. It was clear that the metabolism and synthesis processes of *P. tricornutum* were regulated by MeJA treatment. Furthermore, 12 metabolic pathways related to fucoxanthin synthesis were searched through the KEGG mapper tool in the KEGG metabolic pathway database, where amino acid biosynthesis, carbon metabolism, carbon fixation in photosynthetic organisms, and glycolytic/gluconeogenic metabolic pathways were significantly correlated with the DEGs of MeJA-treated *P. tricornutum*.

Up to now, the critical synthesis steps of fucoxanthin in algae are not clear, which brings great problems for further regulating the synthesis of fucoxanthin in *P. tricornutum*. In this study, a weighted gene co-expression regulatory network was constructed. It was found that the module associated with MeJA treatment was light-green, in which various hub genes were present. Finally, this study took the intersection of DEGs, carotenoid synthesis pathway genes, and members of the light-green module hub genes and discovered overlapping genes. A total of four DEGs related to the fucoxanthin synthesis pathway were identified, among which two genes were significantly up-regulated and two genes were significantly down-regulated, while the up-regulated genes played a key role in the promotion of fucoxanthin synthesis by MeJA treatment in *P. tricornutum*. However, the two up-regulated genes, PHATRDRAFT_54800 and PHATRDRAFT_20677, obtained from the screening have not been studied for gene structure and protein co-function analysis. Therefore, this study is planned to predict the functions of these two genes through sequence alignment and the construction of molecular evolutionary trees with other carotenoid synthesis pathway gene members in *P. tricornutum*. The carotenoid isomerase gene was the last one identified in the synthesis pathway of plant carotenoids and was able to catalyze the conversion of lycopene from cis to trans structure [21]. Currently, carotenoid isomerase has been identified and cloned in a variety of organisms, including corn, tobacco, *Arabidopsis thaliana*, *cyanobacteria*, *Duchenne salina*, and *P. tricornutum* [22,23]. Ma X.’s team has shown that *Zep* was associated with the accumulation of total carotenoids and was up-regulated in mango pulp [24]. Xi W.’s team has found that carnelian blood orange and tangelo orange are rich in total carotenoids, β-carotene, and specific α-carotene, and *Zep* may be a hub gene for carotenoid accumulation in apricot fruits [25]. And this study found that PHATRDRAFT_54800 clustered with Zep family genes, suggesting that PHATRDRAFT_54800 may be functionally similar to *Zep*. Therefore, PHATRDRAFT_54800 may be involved in the synthesis of carotenoid-like metabolites as a carotenoid isomerase, and it may be a core hub gene for promoting the increase of fucoxanthin content by MeJA. In addition, Hornero-Mendez D. et al. reported that NADPH plays an indirect role in the cyclization reaction of carotenoid biosynthesis [26]. In the view of Corpas FJ and others, the plant cells have diverse mechanisms to generate NADPH by a group of NADP-dependent oxidoreductases. NADPH is a key cofactor necessary for cell growth and development, and it is involved in significant biochemical routes such as fatty acid and carotenoid biosynthesis [27]. Zhao X.’s team has concluded that NADH kinase is one of the key sources of NADPH, which has a positive effect on carotenoid biosynthesis [28]. It was believed that another differential hub gene, PHATRDRAFT_20677, was more similar to the NAD(P)-dependent oxidoreductase of the SDR family, which may be involved in the process of synthesizing carotenoid metabolites after MeJA treatment of *P. tricornutum* by acting as a class of NADPH-dependent oxidoreductase.

## 4. Materials and Methods

### 4.1. Cultivation of P. tricornutum

*P. tricornutum* was kindly provided by the Key Laboratory of Marine Biotechnology at Ningbo University, China. Deep, natural seawater was collected and subjected to filtration and autoclaving. After that, the mother liquor of the culture medium was added in the ratio of 1:1000, and *P. tricornutum* in the ratio of 1:10. Culture conditions: the light-dark cycle was 12 h light/12 h dark, light intensity 60 μmol·(m^2^·s)^−1^, and incubation temperature 20 °C. The mass concentrations of MeJA (AR, Sigma, St. Louis, MO, USA) were set at 0 μmol/L, 50 μmol/L, 100 μmol/L, 200 μmol/L, 500 μmol/L, 1000 μmol/L, and 2000 μmol/L, respectively, and 0 μmol/L was regarded as the control group, and the remaining six groups were MeJA-treated groups. Three replicates were set up for each mass concentration.

### 4.2. Determination of Growth Curves of P. tricornutum

After MeJA treatment, 1 mL of *P. tricornutum* was taken at every 24 h interval, and the optical density value at 680 nm of algal cell cultures was measured by an ultraviolet spectrophotometer with three biological replicates. Algal cell densities were calculated according to the optical density regression equation to establish the growth curves of MeJA-treated *P. tricornutum*.

### 4.3. Extraction and Determination of Fucoxanthin

At the time of the maximum biomass of *P. tricornutum*, fucoxanthin was extracted from 80 mL of MeJA-treated algal cultures at different concentrations and centrifuged at 5000 rpm/min for 10 min at 4 °C. With the supernatants discarded, the centrifuged fucoxanthin was freeze-dried for 2 d to obtain its dry weight. Next, the algae were sufficiently ground to powder and then fully dissolved with 1 mL of anhydrous ethanol at 4 °C for 1 h. The fucoxanthin was centrifuged at 5000 rpm/min for 10 min at 4 °C. Furthermore, 1 mL of supernatants was taken for membrane filtration and then stored at 4 °C. Three biological replicates were carried out for each group.

Furthermore, 0.1 mg of fucoxanthin standard (from *Sargassum horneri*) was accurately weighed and diluted multiplicatively with 1 mL of anhydrous ethanol. The concentration of fucoxanthin was identified using high-performance liquid chromatography (HPLC) (YMC-Pack ODS-A, 250 × 4.6 mm, 12 nm, phosphate buffer, flow rate of 1.0 mL/min, wavelength of 445 nm [29]). Regression curves were made by the relations between concentration and cumulative peak area, which were obtained by detecting 10 μL of the substance using HPLC (Waters, Mildford, MA, USA). The fucoxanthin content was determined through the regression curve.

### 4.4. Extraction of Total RNA from P. tricornutum

The MeJA concentration at which the fucoxanthin content was highest was used to treat *P. tricornutum*. When *P. tricornutum* cells were in the logarithm phase, RNA extraction was performed using the plant RNA kit (OMEGA). Total RNA was extracted according to the kit instructions, and cDNA synthesis was subsequently performed using total RNA as a template.

### 4.5. Transcriptome Sequencing

Polymerase chain reaction (PCR) amplification was carried out using the cDNA of *P. tricornutum* as a template. The cDNA was screened with DNA magnetic beads for PCR amplification of the library after the cuttings were recovered, and DNA magnetic beads were used again for the purification of the PCR products to finally obtain the library. After the library construction was completed, the effective concentration of the library was accurately quantified using fluorescent quantitative PCR. After passing the library test, different libraries were mixed according to the effective concentration and the target downstream data volume for sequencing.

### 4.6. Differential Genes Expression Analysis

Differences between the control group and MeJA-treated group were analyzed using the R package “Desea2”, with |Log_2_FC| > 1.2 and FDR < 0.05 as the thresholds. The enrichment analysis of DEGs was performed using GO and KEGG in the R package (v 3.4.3) “clusterProfiler”. Metabolic pathways related to fucoxanthin synthesis were searched through the KEGG mapper tool in the KEGG metabolic pathway database, and the KEGG metabolic pathways related to fucoxanthin synthesis were explored through the R package “clusterProfiler”.

### 4.7. WGCNA

Expression profiles of the control and MeJA-treated groups were preprocessed to match the expression matrices of the differential genes with their gene names, followed by counting the expression values of the samples by the formula Log(CPM + 1). The minimum number of genes in the WGCNA module was set to 30, the height of the clustering tree was cut to 0.3, and similar modules were merged with a cutting height of 0.25. Cytoscape in the R package (v 3.4.3) “WGCNA” was used to draw gene-based network diagrams. The hub genes within the modules were screened for |kME| > 0.9. DEGs, carotenoid biosynthesis pathway (ko00906 carotenoid biosynthesis) genes, and members of the MeJA-related module hub genes were intersected to obtain the DEGs of the carotenoid synthesis pathway related to MeJA.

### 4.8. Functional Analysis of Key Genes for Fucoxanthin Synthesis

Sequence alignment analysis of the carotenoid synthesis pathway was performed using the basic local alignment search tool (BLAST) with a critical E value of 0.0001. The neighbor-joining method was used to calculate the molecular evolutionary relationships of the genes related to the carotenoid synthesis pathway. The bootstrap value was then employed as a quality control criterion for the branching relationship of the evolutionary tree, and the branches with a bootstrap value < 0.5 were collapsed. The molecular evolution tree data were saved.

## 5. Conclusions

In summary, the carotenoid synthesis-related genes PHATRDRAFT_54800 and PHATRDRAFT_20677 are the key genes for the promotion of fucoxanthin synthesis in MeJA-treated *P. tricornutum*. PHATRDRAFT_54800 may be a carotenoid isomerase, while PHATRDRAFT_20677 may be involved in fucoxanthin synthesis in the MeJA-treated *P. tricornutum* through exerting the role of the SDR family NAD(P)-dependent oxidoreductase. In this study, the effects of MeJA on cell growth and the fucoxanthin content of *P. tricornutum* were explored, which provided more evidence for marine pharmacy. The specific mechanism of the promotion of fucoxanthin synthesis with the MeJA-treated samples needs to be further explored in the future so as to develop other potential biological activities of fucoxanthin. In addition, there are some limitations in this study; only two unknown genes are roughly inferred, but the function of unknown genes and the mechanism of how they affect fucoxanthin synthesis remain to be discovered.

## Data Availability

Data are contained within the article.

## Supplementary Materials

The following supporting information can be downloaded at: https://www.mdpi.com/article/10.3390/md22010007/s1, Table S1. The top 10 similar sequences were obtained by BLAST sequence alignment.
