# Supplementary material for: Identification of Potential Factors for the Promotion of Fucoxanthin Synthesis by Methyl Jasmonic Acid Treatment of Phaeodactylum tricornutum
Source: Mar Drugs. 2023 Dec 20;22(1):7. doi: 10.3390/md22010007 (PMC10817275; doi:10.3390/md22010007)
Supplement: Supplementary file 1 [file marinedrugs-22-00007-s001.zip › marinedrugs-2718074-supplementary.pdf]

Table S1. The top 10 similar sequences obtained by BLAST sequence alignment.

Describe	Species	Score	Similarity	E value
Predicted protein	*P. tricornutum CCAP 1055/1*	481	100%	$2.00E^{-171}$
SDR family NAD(P)-dependent oxidoreductase	*Oceanibium sediminis*	248	100%	$1.00E^{-79}$
SDR family NAD(P)-dependent oxidoreductase	*Marinobacter sp. PJ-16*	232	98%	$3.00E^{-73}$
short-chain dehydrogenase	*Marinobacter sp. B9-2*	231	98%	$6.00E^{-73}$
SDR family NAD(P)-dependent oxidoreductase	*Marinobacter sp. NP-4 (2019)*	229	98%	$7.00E^{-72}$
SDR family NAD(P)-dependent oxidoreductase	*Persicimonas caeni*	228	98%	$2.00E^{-71}$
SDR family NAD(P)-dependent oxidoreductase	*Persicimonas caeni*	228	98%	$2.00E^{-71}$
TPA: SDR family NAD(P)-dependent oxidoreductase	*Marinobacter sp. nov.*	227	98%	$5.00E^{-71}$
SDR family NAD(P)-dependent oxidoreductase	*Marinobacter sp. ELB17*	226	98%	$1.00E^{-70}$
SDR family NAD(P)-dependent oxidoreductase	*Marinobacter sp. BSs20148*	226	98%	$1.00E^{-70}$
